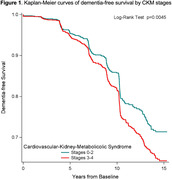# Cardiovascular‐Kidney‐Metabolic Syndrome and Incidence of Dementia among Older Adults

**DOI:** 10.1002/alz.090626

**Published:** 2025-01-09

**Authors:** Xiaqing Jiang, Amber L Bahorik, Christina S. Dintica, Kristine Yaffe

**Affiliations:** ^1^ University of California, San Francisco, San Francisco, CA USA; ^2^ San Francisco Veterans Affairs Health Care System, San Francisco, CA USA; ^3^ Departments of Psychiatry and Behavioral Sciences, Neurology, and Epidemiology, University of California San Francisco, San Francisco, CA USA

## Abstract

**Background:**

Cardiovascular‐Kidney‐Metabolic Syndrome (CKM), a systemic interplay among metabolic risk factors, chronic kidney disease (CKD), and cardiovascular disease (CVD), has profound impacts on cardiovascular events and mortality, yet its association with dementia risk remains poorly understood. With data from the Health, Aging, and Body Composition study, we investigated the association between CKM and dementia risk among older adults.

**Method:**

We studied 2,722 participants (mean age 74 ± 2.8, 53% female, 37% Blacks) who had measurements for CKM and were free of cognitive impairment at baseline (1997‐98). We defined incidence dementia over 15 years based on hospital records, prescription for dementia medication, or a decline ≥1.5 standard deviations below the mean on the Modified Mini‐Mental State (3MS) score. CKM staging, as defined by the American Heart Association, was based on constructs comprising dysfunctional adiposity, metabolic risk factors, CKD, and CVD. We used cause‐specific hazards models to assess the association between CKM, both as a linear term and dichotomously (CKM stage 3‐4 vs. 0‐2), and time to dementia diagnosis, treating death as censored. Models were adjusted for age, sex, race, education, and baseline 3MS.

**Result:**

Participants with advanced CKM stages (3‐4) were prevalent at baseline: 3% stage 0 (no CKM risk factors), 4% stage 1 (excess or dysfunctional adiposity), 34% stage 2 (metabolic risk factors), 17% stage 3 (subclinical CVD in CKM), and 43% stage 4 (CVD in CKM). When modeled as a linear term, a greater CKM stage is significantly associated with an 11% increase in the risk of incident dementia (hazard ratio [HR] 1.11, 95% CI 1.02 to 1.21) after multivariable adjustment. Compared to participants with CKM stages 0‐2, those with CKM stages 3‐4 had a significant 24% increase in the risk of incident dementia (HR 1.24, 95% CI 1.03 to 1.50) after multivariable adjustment. Sensitivity analyses indicated no significant interaction between CKM and race, sex, or *APOE ε4* (p‐values>0.05).

**Conclusion:**

Among community‐dwelling older adults, CKM is associated with increased risk of developing dementia. Older adults with CKM may need to be followed closely for adverse cognitive outcomes, and modifiable risk factors for CKM need to be managed adequately.